# Characteristics and outcomes of patients with primary abdominopelvic aggressive angiomyxoma: a retrospective review of 12 consecutive cases from a sarcoma referral center

**DOI:** 10.1186/s12893-023-01974-z

**Published:** 2023-04-12

**Authors:** Wenjie Li, Jun Chen, Enlong Zhang, Weida Chen, Yuru Hu, Chengli Miao, Chenghua Luo

**Affiliations:** 1 Department of Retroperitoneal Tumor Surgery, Peking University International Hospital, Beijing, 102206 China; 2 Department of Radiology, Peking University International Hospital, Beijing, 102206 China

**Keywords:** Aggressive angiomyxoma, Clinicopathological feature, Outcome, Surgical treatment, Recurrence

## Abstract

**Background:**

Aggressive angiomyxoma (AAM) is a rare mesenchymal tumor that mostly arises from the pelvic and perineal soft tissues. Few studies reported its characteristics and outcomes previously due to its rarity and challenges of treatments. This study aimed to investigate the clinical characteristics as well as surgical and short-term survival outcomes of primary abdominopelvic AAM.

**Methods:**

Medical records of patients who were admitted to surgery with pathological confirmation of primary abdominopelvic AAM at Peking University International Hospital from January 2016 through December 2021 were retrospectively retrieved from our retroperitoneal tumor database. Demographics, operative outcomes and pathological findings were collected. Patients received followed-up routinely after the surgery. Survival probabilities were calculated and determined through Kaplan–Meier analysis.

**Results:**

A total of 12 consecutive patients (male/female 4:8) were included in this study. The median age was 45 years old. The clinical presentation varied among individuals, consisting of 2 abdominal discomforts, 4 constipations, 1 lumbago, 1 prolonged menstruation, and 1 buttock swelling. R0/R1 resection was achieved in 100% of patients. Postoperatively, 50% of patients developed various complications including 3 fistulas and 3 wound infections. No operative mortality was observed. Histopathology of all patients was suggestive of AAM. Immunohistochemistry was done with a 91.7% positive rate for estrogen and progesterone receptors. The median recurrence-free survival time was 38 months. There were no cases of deceased or presented with distal metastasis during a median of 42 months’ follow-up.

**Conclusions:**

The clinical manifestations of abdominopelvic AAM are mostly atypical. Surgical resection with curative intents remains the mainstay treatment of this disease, which was strongly suggested in experienced sarcoma centers due to the high probability of severe postoperative complications. In addition, long-term follow-up is necessary due to the high rate of local recurrences.

## Introduction

Aggressive angiomyxoma (AAM) is a very rare locally aggressive soft tissue tumor that was first described by Steeper and Rosai in 1983, with less than 350 cases reported to date [1]. Due to its rarity and atypical clinical presentation, it often occurred in the pelvic-perineal region mimicking other soft tissue diseases as mucinous liposarcoma, malignant mucinous fibrous histiocytoma and angiomyofibroblastoma [2, 3]. At present, neither comprehensive studies focusing on the diagnosis, treatment and prognosis of AAM were introduced, nor consensus in this area was arrived. Knowledge of characteristics, treatment options were just acquired from few small case series reports previously [4, 5]. The aim of this study was to explore the clinical characteristics, surgical and survival outcomes of a single-institution cohort of abdominopelvic AAM patients undergoing surgeries.

### Patients and methods

All consecutive patients with abdominopelvic AAM undergoing surgical resection from January 2016 through December 2021 at Peking University International Hospital were retrospectively reviewed. Patients who underwent surgeries for the first time with pathological findings of AAM were included in this study. Patients with recurrent lesions or histories of second primary malignant tumors were excluded.

### Ethics approval

The study design was reviewed and approved by the Institutional Review Board of Peking University International Hospital (No.2019–032 (BMR)). All procedures performed in this study were in accordance with the ethical standards of the institutional and national research committee and with the 1964 Helsinki Declaration and its later amendments.

### Data collection

Data were collected prospectively through our retroperitoneal tumor database, including demographics, clinical presentations, preoperative labs and images, operative outcomes, as well as pathological findings. Patients were followed-up routinely through telephone or clinic visits.

### Statistical analysis

Categorical variables were reported as frequency (%) and continuous variables were reported as mean with standard deviation (SD) or median with interquartile range (IQR). The follow-up period and survival times were right censored using September 30, 2022 as the cut-off date. Recurrence free survival were estimated by Kaplan–Meier method. A *P* value of less than 0.05 was considered statistically significant. Software of STATA Version 14.0 was used for statistics.

## Results

### Demographics and clinical manifestations

A total of 12 consecutive patients (male/female 4:8) were included in this study. The median age was 45 years old. The clinical presentation varied among individuals. Demographics and characteristic details were listed in Table 1. Of whom, 3 were asymptomatic and found occasionally from abdominopelvic CT scans. The interval between initial diagnosis and treatment ranged from 0.5 months to 14 years. None of cases had a family history of soft tissue sarcoma or severe comorbidities previously.

**Table 1:** Demographics and characteristics of AAM in all 12 patients

Variables	N	% or range
Total	12	(100)
Gender (male)	4	(33.3)
Median age at surgery (year) (range)	45	(27–66)
Median interval between diagnosis and surgery (month) (range)	6	(0.5–168)
Chief Complains		
Abdominal discomfort	2	(16.7)
Constipation	4	(33.3)
Lumbago	1	(8.3)
Prolonged menstruation	1	(8.3)
Buttock swelling	1	(8.3)
Asymptomatic	3	(25)
Nutrition index (mean ± SD)		
Haemoglobin (g/L)		134 ± 6.0
Albumin (g/L)		39.3 ± 2.1
BMI (kg/m^2^)		21.3 ± 4.3
Median tumor size (cm) (range)	15	(4–30)
Tumor location		
Retroperitoneal	1	(8.3)
Abdominal-pelvic region	1	(8.3)
Trans-pelvic extended to perineal area	10	(83.3)

### Preoperative labs, images and biopsies

**Fig. 1:**
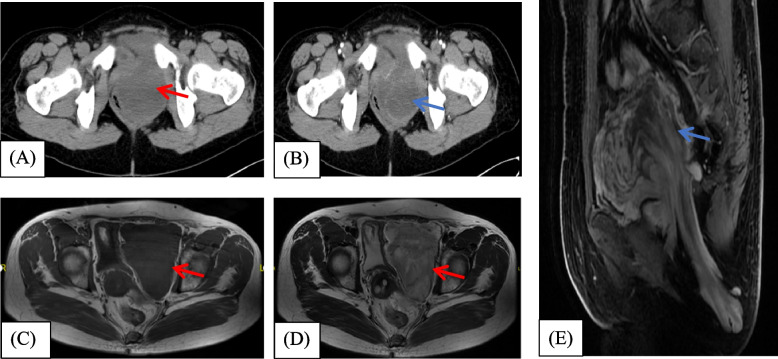
CT images of AAM (case11) The lesion was located in the left para-rectal space (Red arrow). **A** Axial plain CT scan showed lower density of the lesion compared with density of the muscle. **B** Axial enhancement CT scans showed the lesions are mildly enhanced and appear ‘layered’ (Blue arrow). The mass was clearly bounded, and the rectum and uterus were displaced to the right. MRI images of AAM (case7) A soft tissue mass was seen in the left pelvis with the lesion extending through the pelvic floor to the subcutaneous fat of the left buttock. The lesion was well defined and the surrounding structures were displaced (Red arrow). **C** Axial unenhanced T1-weighted MRI shows a mass with isosignal relative to the muscle. **D** Axial T2-weighted MRI showed the mass as a mixed signal with predominantly high signal. **E** Sagittal enhanced T1-weighted MRI showed the swirling and layering pattern of marked enhancement within the mass (Blue arrow)

Lab tests including blood count tests, biochemistry and tumor markers preoperatively. The median hemoglobin, albumin levels were 134 g/L, 39.3 g/L, respectively. Tumor markers like AFP, CA199, CA125, and CEA were in normal range in all patients. About imaging, 11 cases underwent plain and contrast-enhanced CT scan and 5 cases received magnetic resonance imaging (MRI) with and without contrast. Typical plain CT scan showed a significant lower density of tumor compared with muscle (Fig. 1A). In contrast-enhanced CT scan, 7/12 patients showed ‘swirled’ or ‘layered’ signs which could be considered as unique features (Fig. 1B). MRI showed low intensity on T1 and high intensity on T2-weighted images. The ‘swirled’ or ‘layered’ signs could also be seen on contrast-enhanced scan in 4/5 cases (Fig. 1C, D, E). Besides, 6 patients underwent preoperative needle biopsy suggesting 4 atypical spindle cell tumors, 1 leiomyosarcoma and 1 well-differentiated liposarcoma.

### Operative outcomes

Each patient’s surgical plan was determined after retroperitoneal tumor multidisciplinary team (RPT-MDT) discussion. All procedures were performed by the same surgical team. Surgical approaches were defined and categorized per location of the tumor, including 7 abdominal approaches (AA), 3 sacrococcygeal perineal approaches (SPA), as well as 2 abdomino-sacral approaches (ASA). The mean operative time was 260.8 ± 108.2 min. The median intraoperative blood loss was 500 ml (IQR 250,1400 ml). The overall postoperative rate was 50% (6/12). The details of operative outcomes were shown in Table 2. All patients were discharged safely with no operative mortality.

**Table 2:** Operative outcome of AAM patients listed case by case

Cases	Age/gender	Symptom	Size(cm3)	Location	Approach	OPT(min)	IBL (ml)	Complication
1	27/F	Asymptomatic	18 × 10 × 4	TPP	SPA	150	300	Rectal-vaginal-perineal fistula
2	49/F	Abdominal discomfort	20 × 12 × 30	AP	AA	300	1500	None
3	30/F	Asymptomatic	16 × 9 × 3.5	TPP	AA	290	500	None
4	46/F	Constipation	12 × 7 × 5	TPP	SPA	120	50	None
5	31/M	Abdominal discomfort	10 × 6 × 5.5	TPP	AA	160	200	None
6	57/M	Low back pain	4 × 3.8 × 2	RP	AA	150	300	None
7	46/F	Buttocks swelling	11 × 9 × 5	TPP	SPA	160	100	Wound infections
8	49/M	Constipation	12 × 9 × 3	TPP	ASA	390	1200	Wound infections
9	27/F	Constipation	10 × 8 × 6	TPP	AA	390	1500	None
10	34/F	Prolonged menstruation	13 × 10 × 3	TPP	AA	300	500	Rectal-vaginal-perineal fistula
11	44/F	Constipation	27 × 24 × 5.5	TPP	ASA	300	1300	Wound infections
12	66/M	Asymptomatic	20 × 18 × 4	TPP	AA	420	3000	Rectal-perineal fistula

*OPT* Operation time, *IBL* Intraoperative blood loss, *TPP* Trans-pelvic diaphragm, extended to perineal area, *AP* Abdominal-pelvic region, *RP* Retroperitoneal region, *AA* Abdominal approach, *SPA* Sacrococcygeal perineal approach, *ASA* Abdomino-sacral approaches

### Histopathological outcomes

**Fig. 2:**
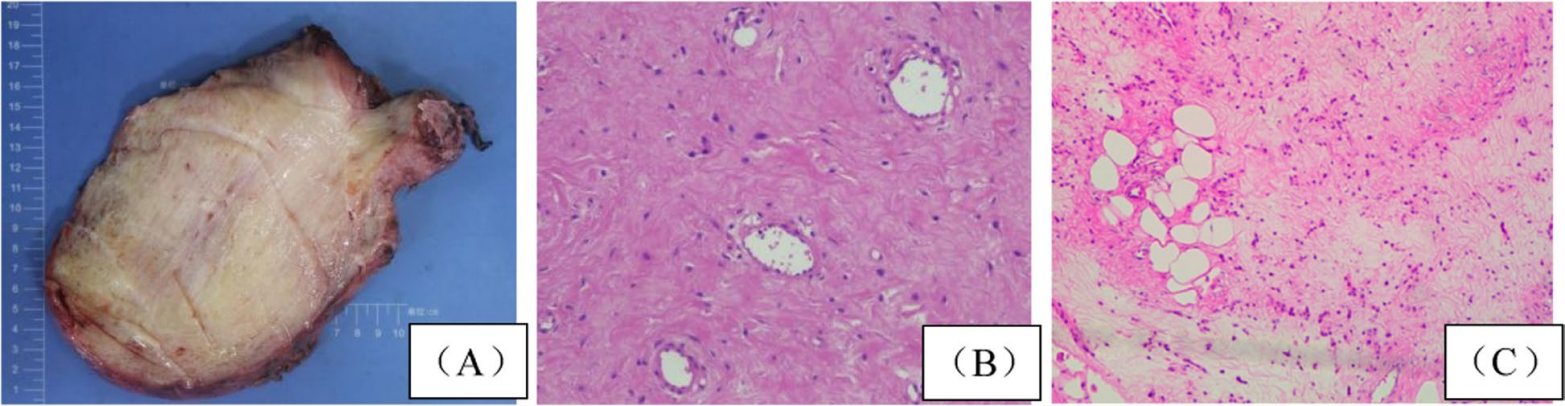
Pathological manifestations of AAM. **A** Gross appearance of AAM in Case 11. The cut surface was grey-white and soft, with incomplete tumor envelope. **B** Short spindle-shaped or stellate tumor cells were sparsely distributed with a milder morphology. The interstitium of the tumor contained abundant blood vessels(case11) (HEx100). **C** The tumor invaded the surrounding adipose tissue (case2) (HEx100)

Generally, AAM is often large in size and has a spherical or lobulated gross appearance pathologically. In this group, 6 tumors were surrounded with intact envelopes while other 6 were incomplete. The cut surface of the masses presented as grey-white, grey-red or jelly-like (Fig. 2A). Microscopic examination revealed that the tumor cells were sparsely located with asteroidal or short spindle shapes. Cell morphology was mild and the nuclear schizogony was rare (Fig. 2B). The marginality was hardly determined and poorly demarcated from the surrounding adipose tissue, with normal nerves and blood vessels locally seen to be encapsulated (Fig. 2C). Immunohistological examination were conducted in all cases. Estrogen and progesterone receptors were diffusely positive in the 91.7% (11/12) of cases microscopically. Other predominate biomarkers like smooth muscle actin (SMA), CD34, desmin were listed in Table 3.

**Table 3:** Immuno-histochemical staining profiles of 12 AAM patients

Antigen	N of Positive (12)	Positive rate (%)
ER	11	91.7
PR	11	91.7
SMA	8	66.7
CD34	10	83.3
S-100	2	16.7
Desmin	12	100

*ER* Estrogen receptor, *PR* Progesterone receptor, *SMA* Smooth muscle actin

### Follow-up and survival outcomes

In our study, none of the patients received adjuvant treatments, including, but not limited to hormones, radiotherapies, or chemotherapies postoperatively. All patients were followed up routinely through telephone interviews, clinic visits. 1 patient was lost to follow-up. The median follow-up time was 42 months (range 10–74 months). Locally recurrence occurred in two cases (18.2%), determined by images. The median recurrence free survival time was 38 months. There was no distant metastasis observed postoperatively. And all patients were alive with favorable quality of life at the last follow up on September 2022.

## Discussion

AAM is commonly considered as a rare benign tumor originated from mesenchymal tissues. However, local recurrence could be observed in previous case reports, whereas it has an extreme low tendency to metastasize with only 3 cases reported to date [6, 7]. The pathogenesis of this tumor has not been well elucidated. Recent studies highlighted the notion that the involvement and misexpression of HMGA2 might be associated to development of the disease [8, 9]. The lesion was often located at the pelvis and perineum area. It rarely aroused from the spermatic cord, testes or sciatic rectal fossa. It occurred more often in female than male patients, especially at their reproductive ages [10]. In this study, the ratio of male to female was 1:2, with 50% of women in their reproductive years. There is no specific clinical manifestation of the tumor in its early stage, showing a slow and insidious growth pattern. The progression could last for decades before it leads to some symptoms. About 25% of patients in this study visited the hospital for getting consultation of asymptomatic lesions occasionally found during physical examination. In case 3, a 2-cm mass found in the pelvis on physical examination previously, was enlarged to 10 cm on repeat CT scan 2 years later with no complains. Rest patients in this group also presented with atypical symptoms like abdominal discomforts, constipations, lumbago, prolonged menstruation and buttock swelling.

The preoperative diagnosis of AAM was relied on images and needle biopsy. ‘Layered’ or ‘swirled’ sign was considered as a typical signal in diagnosis of AAM in CT or MRI scans, which was thought to be related to abundant collagen fibers and vascular structures contained in the tumor. It was presented in 63.6% (7/11) of CT and 80% of MRI scans in this case series. Besides, cystic components and necrotic hemorrhage may be present in the tumor parenchyma, showing a significant lower density of the lesion in plain CT and progressive enhancement on contrast CT scans, compared to the density of the muscle density. This is consistent with what is described in the literature [11]. These characteristics were also helpful in the differentiate diagnosis from other subtypes of soft tissue tumors [12, 13]. Mucinous liposarcoma often had cystic changes, calcifications and adipose components in images, which could be easily differentiated from AAM, whereas imaging of malignant mucinous fibrous histiocytoma often disclosed a regular pattern with cystic haemorrhage and necrosis in plain CT and progressive or persistent enhancement in enhanced CT, while the ‘layered’ signs are rare to see. Angiomyofibroblastoma was also a type which was difficult to distinguish from AAM from images. It was often capsulated by a rounded, well-defined border with remarkable enhancement signs instead of the typical ‘swirled’ signs. A needle biopsy might be helpful in determining a diagnosis [14]. However, microscopic verification of AAM can be extremely challenging due to small specimen acquired, atypical tumor cell proliferation and a lack of immune-histological tumor markers. Thus, it is not recommended as a routine process, per suggestions and consensus from TARPSWG (Transatlantic Australasian Retroperitoneal Sarcoma Working Group) due to its limited value. Even though four out of six patients in this series showed spindle cell tumors in the biopsy, a direct preoperative diagnosis of AAM could not be made, let alone the two misdiagnoses of leiomyosarcoma or liposarcoma. Therefore, the preoperative diagnosis of AAM remains a significant challenge that requires further exploration of common features or specific tumor biomarkers in larger series.

Currently, surgical resection remains the mainstay treatment of AAM. Locally extended resection of tumor with surrounded barriers achieving clean margins is the key to decrease the tumor recurrence [15]. Patient with clean margins was reported as an independent factor significantly associated with longer progression free survival. A retrospective study with more than 100 cases however refuted this belief and disclosed that patients with clear resection margins didn't bring with lower recurrences. It showed no statistical difference in residual disease-free period between the groups of patients with positive and negative resection margins (40% versus 50% at 10 years) [16]. And in no doubt, it is challenging to achieve clean margins in these cases due to its nature of large size, deep anatomical site, aggressive pattern, important physiological functions domination. Tumor involving with urethra, vagina, rectum and anal sphincter would lead the surgery being extremely difficulty. In this study, the maximum diameter of tumor was up to 25 cm. Almost half of patients developed various complications after a median of 6 h-operation. Thus, we need balance complete resection intention against with high probabilities of severe morbidity in each individual case. In some scenarios, incomplete resection is acceptable when the incidence of surgical complications is high or fertility is expected to be preserved. Typically, the primary surgical procedures involve the following steps: 1) making a midline incision to open the abdominal-pelvic cavity; 2) dissecting the intestine to expose the pan-pelvic area and the tumor; 3) isolating or detaching the rectum and its mesentery, bladder, uterus, and ovary from the surface of the tumor, if possible; 4) exposing the retroperitoneal and presacral spaces to isolate the tumor from the pelvis; and 5) removing the tumor after isolating it from surrounding tissues. Organ resections, with or without reconstruction, may be necessary if the tumor has infiltrated the organs. To decrease the morbidity and mortality, surgical policies and advices were applied as: 1) Appropriate surgical approach based on tumor size and location. Abdominal approach was the most frequently adopted (10 patients). It is the best as well as the most familiar and easiest way for removing abdominal-pelvic tumors from anterior side. As to tumors protruded to the perineal and sacral space, sacrococcygeal-perineal or abdomino-sacral were better options which could provide good vision and tumor exposure. In addition, incision enlargement and extension could be managed more flexibly without impairing anal and urinal functions, despite of higher possibilities of fistulas (2 recto-vaginal-perineal fistulas and 1 rectal-perineal fistula) developed after surgery. 2) Prophylactic ureteral catheter placement (PUCP). PUCP was advocated as an effective way to prevent ureteral injuries by colorectal surgeons, urologists, and gynecologists despite of no evidence-based guidelines for further validation. PUCP is more likely being recommended in surgeries involving retroperitoneal and pelvic area due to its aids on identification and guidance of ureter based on our previous case reports of retroperitoneal liposarcoma [17]. All 5 patients who underwent PUCP in this study as well, had no postoperative ureteral fistula or stricture. 3) Effective and longer duration of drainage tube placement. Drainage tube placed in right position was utmost important in surgeries since anterior sacral cavity left post tumor resection was normally large and irritated. Negative pressure suction, longer duration of drainage tube placement and continuous irrigation might be helpful in decreasing the possibilities of abdominal collections and infectious. No abdominal or pelvic collections were observed in this study after a median of 14d’s duration of tube. 4) Vascular embolization and balloon occlusion. These two strategies were also been considered in bleeding control in retroperitoneal and pelvic surgeries especially in rich-blood-supply tumor and tumors with major vessels involvements [18, 19].

Pathologically, mitosis was rare to seen in these cases and no specific immune-histological markers were validated in AAM, which is generally consistent with previous literature [20]. Tumor proliferation index like Ki-67 is lower to 2% indicating that radiotherapy and chemotherapy might not be helpful. Recurrence rate of AAM ranges from 25 to 47% with approximately 85% recurring within 5 years of surgery. Distant metastases from this disease are extremely rare, but the prognosis is good [9]. And in this group, no cases received adjuvant therapy postoperatively. Recurrence occurred in two cases (18.2%) at 31 and 8 months after surgeries. No case developed with distant metastasis. Besides, AAM is a likely hormone dependent tumor [21, 22]. Estrogen receptor (ER) and progesterone receptor (ER) positive were confirmed in 91.7% (11/12) of patients (including males). In addition, Gonadotropin-releasing hormone agonists (GnRH-a), Aromatase inhibitors (AIs) and Selective estrogen receptor modulators (SERM) were also used with great success in preoperative neoadjuvant therapy or treatments for recurrences [6, 23, 24]. Reminding that, they should not be used in patients who are ER, PR receptor negative due to its side effects as menopausal symptoms, bone loss, fatigue and weight gain. Therefore, the optimization of hormone therapy is still need to be validated by further researches.

This study has its intrinsic limitation due to its retrospective nature with small size. Statistical biases were inevitable and not well calculated. And the median follow-up time was short. Well-designed prospective study and multi-centered data collection are needed to better understand AAM.

## Conclusions

Primary aggressive Angiomyxoma is a rare benign neoplasm with a locally infiltrative behavior, localizing in pelvic and perineal regions. The clinical presentation is atypical and the preoperative diagnosis relies on radiologic images. Wide surgical resection is the gold standard treatment. Although it shows local recurrence, metastasis is extremely rare. Considering the fact that it has a high tendency for local invasion, long-term follow-up remains the best course of management.

## Data Availability

The datasets used and analyzed during the current study are available from the first author on reasonable request.

## Abbreviations

AAM: Aggressive angiomyxoma
AA: Abdominal approach
SPA: Sacrococcygeal perineal approach
ASA: Abdomino-sacral approach
OPT: Operation time
IBL: Intraoperative blood loss
TPP: Trans-pelvic diaphragm, extended to perineal area
AP: Abdominal-pelvic region
RP: Retroperitoneal region
ER: Estrogen receptor
PR: Progesterone receptor
